# ANTIBACTERIAL EFFECTS OF SINGLE AND COMBINED CRUDE EXTRACTS OF *SYNADENIUM GLAUCESCENS* AND *COMMIPHORA SWYNNERTONII*

**DOI:** 10.21010/Ajid.v16i2S.2

**Published:** 2022-08-17

**Authors:** Ochollah G. Mary, Msengwa S. Zaituni, Mabiki P. Faith, Kusiluka J.M. Lughano, Mdegela H. Robinson, Olsen E. John

**Affiliations:** 1 Department of Chemistry and Physics, College of Natural and Applied Sciences, Sokoine University of Agriculture, P.O. Box 3038, Morogoro, Tanzania; 2 Mzumbe University, P.O. Box 1, Morogoro, Tanzania; 3 Department of Veterinary Medicine and Public Health, Sokoine University of Agriculture, P.O. Box 3015, Morogoro, Tanzania; 4 Department of Veterinary and Animal Sciences, University of Copenhagen, Stigbøjlen 4, Frederiksberg C, Denmark

**Keywords:** *Synadenium glaucescens*, *Commiphora swynnertonii*, Antibacterial activity, Synergism, Antagonism, Additive and Crude extracts

## Abstract

**Background:**

*Synadenium glaucescens and Commiphora swynnertonii* are among the reported plants used traditionally for treatment of bacterial infections. This study reports antibacterial effects of single and combined extracts from leaves, stem and root barks of *Commiphora swynnertonii* and *Synadenium glaucescens*.

**Materials and Methods:**

Plants were collected from Manyara and Njombe regions in Tanzania. Extraction was done using dichloromethane and methanol. The extracts were assessed for antibacterial activity against Gram-positive bacteria (*Staphylococcus aureus* and *Enterococcus faecalis*) and Gram-negative bacteria (*Escherichia coli*, *Klebsiella pneumonia* and *Pseudomonas aeruginosa*). Minimum Inhibitory Concentrations (MIC) was determined by broth microdilution, while Fractional Inhibitory Concentration (FIC) indices were calculated from MIC values of combined extracts to determine combination effects.

**Results:**

Strong antibacterial activities were demonstrated by all extracts of *S. glaucescens* (MIC 0.011-0.375mg/mL) against Gram-positive bacteria and methanol extracts of *C. swynnertonii* (MIC 0.047-0.375mg/mL). Synergistic effect was observed when combining methanol extracts of *C. swynnertonii* stem bark with *S. glaucescens* leaves against *S. aureus* (∑FIC 0.5), Other synergistic effects were observed against *E. faecalis* with dichloromethane extracts of *C. swynnertonii* stem bark and *S. glaucescens* stem bark (∑FIC 0.5), and *C. swynnertonii* root bark and *S. glaucescens* root bark (FIC index 0.3). For the remaining combinations, mainly additive effects were observed.

**Conclusion:**

Synergistic effects on bacteria were observed by combining different plant parts of S*. glaucescens and C. swynnertonii* suggesting that it could be beneficial to combine such extracts when used for antibacterial purposes.

## Introduction

Herbal products have been used as medicines since the commencement of human life (Masimba *et al.*, 2014). The recipes for medicinal plant preparation for the treatment of several ailments are evidenced from the earliest Sumerian, Indian, Egyptian, and Chinese publications (Karunamoorthi *et al.*, 2013). Unlike pharmaceuticals, where the ingredients are well defined and characterized, herbal products contain multiple bioactive compounds with little or no understanding of how these compounds function, likewise the effect of herbal combinations is usually poorly characterized (Gupta *et al.*, 2017). When herbal combinations are administered together there is a possibility of causing chemical or pharmacological effects that may increase or decrease the effectiveness or severity of adverse effects via synergistic, additive, or antagonistic effects (Shi and Klotz, 2012; Sheng *et al.*, 2018). In Tanzania, people access a variety of medicines to meet their healthcare needs. At least 70% of the population is estimated to use traditional medicines (Stanifer *et al.*, 2015). *Synadenium glaucescens* (Mvunjakongwa in Swahili) and a tropical tree *Commiphora Swynnertonii* (Oltemwai in Maasai) which belong to the families *Euphorbiaceae* and *Burseraceae* respectively are among the medicinal plants used by Tanzanians to treat various diseases in humans (Bakari *et al.*, 2012; Mabiki *et al.*, 2013; Mkangara *et al.*, 2014). These plants contain secondary metabolites such as alkaloids, flavonoids, phenols, terpenoids, anthraquinones, steroids, and essential oils (Mabiki *et al.*, 2013; Kalala *et al.*, 2014). Such compounds are reported to have activity against infections caused by bacteria, fungi, viruses, and pests in humans and livestock (Bakari *et al.*, 2012; Mabiki *et al.*, 2013; Mkangara *et al.*, 2014). Despite the exhibited potentials of some individual herbal drugs in the treatment of some infectious diseases, there are reported failures of most single drugs or medicines in the treatment of many pathogenic infectious diseases (Wang *et al.*, 2021). The root causes of these hindrances are reported to be the development of anti-microbial resistance, a narrow antimicrobial spectrum, and limited activity of antimicrobials agents (Rubaka *et al.*, 2014; Ayukekbong *et al.*, 2017)**.** As a result, these failures may cause an increase in the number of morbidities, mortality, disability, and socioeconomic costs (Stanifer *et al.*, 2015). Therefore, there is a need for the search for novel antibacterial drugs from natural resources like herbs to combat the reported hindrances for antimicrobial activities (Bhardwaj *et al.*, 2016). Due to synergistic effects resulting between the combination of more than one drugs in the treatment of microbial infections, it has been reported to be the best techniques to fight against hindrances for antimicrobial effects (Vuuren and Viljoen, 2011). Hence, this study focused on evaluation of antibacterial activities of combined extracts from leaves, stem barks, and root barks of *S. glaucescens* and *C. swynnertonii*. The results from this study, especially for the combinations which demonstrated synergistic effects, may be adopted for the treatment of bacterial infections. However, further study on safety for these combinations is highly recommended.

## Materials and Methods

### Study design and study Area

This study was an experimental one where the antibacterial effects of combinations of herbal medicines were assessed based on their effects and efficacies against selected bacteria. The study was conducted in the chemistry laboratory, Department of Chemistry and Physics, and microbiology laboratory, Department of Biosciences, of the College of Natural and Applied Sciences of the Sokoine University of Agriculture (SUA).

### Plant collection and preparation

The leaves, stem, and root barks of *Synadenium glaucescens* were collected from Mtulingala village in Njombe region coordinates 08^o^34’ to 08^o^49’ S and 08^o^34’ to 03^o^55’ E meters above sea level. The root barks, leaves, and stem barks of *Commiphora swynnertonii* were collected from Mirerani-Simanjiro District in Manyara region coordinates 03^o^36’ to 03^o^14.73’ S and 36^o^50’ to 36^o^18.05’ E meters above the sea level. Plant parts were washed with clean water then peeled to separate the barks and wood. Plant materials were dried in a dark room at 20^o^C at the Tanzania Tree Seed Agency Laboratory, Morogoro. Dry samples were grounded separately using a lab mill machine (Christy Hunt Engineering Ltd, England) to obtain approximately 2mm particle size. The selection of these plant parts was based on the previously conducted studies on antimicrobial activity against selected bacteria (Max *et al.*, 2014; Mkangara *et al.*, 2014).

### Reagents

Solvents used for extraction and dissolving sample in this study were methanol (Finer Chemical, Gujarat-India), dichloromethane, and dimethyl Sulphoxide (Loba Chemie, Mumbai-India). The standard antibiotic used as positive control was gentamicin (Sigma-Aldrich, Germany).

### Extraction and Concentration

Extraction of extracts were carried out using the method used by Bakari *et al*. (2012) and Max *et al*. (2014)**.** Briefly, 1000g of dry ground plant materials were extracted by dichloromethane using hot continuous extraction method at 50^o^C for 4 hours whereby 33g of dry ground samples were injected into each thimble (33mm diameter, 80mm length) and extracted using Soxhlet apparatus. The samples were filtered and the obtained solid residues were soaked in methanol at room temperature (25-30^o^C) for 72 hours. All samples were filtered using Whatman No.1 filter paper (Maidstone-Kent, UK). The filtrates were concentrated in a rotary evaporator (Buchi Labortetechnik, Flawil, Switzerland) with a bath maintained at 40^o^C. The obtained crude extracts were air-dried to remove remains of solvents. The Dried extracts were stored in a refrigerator at 6 ^o^C until further use.

### Test bacterial strain

Gram-positive bacteria used were *Staphylococcus aureus* American Type Culture Collection (ATCC 29213) and *Enterococcus faecalis* (ATCC 51559). Gram-negative bacteria used were *Escherichia coli* (ATCC 25922), *Klebsiella pneumonia* (ATCC 1145), *Pseudomonas aeruginosa* (ATCC 27853). These belong to species that are major causes of nosocomial infections, and where antimicrobial resistance is a high treat to human health (WHO, 2002).

### Preparation of individual and combined crude extracts solutions

A stock concentration of 3 mg/ml crude extract from leaves, stem barks and root barks of *S. glaucescens* and *C. Swynnertonii* was made. Depending on the MIC value of each crude extract, the different concentrations were made to make working bench solutions. The extracts were combined in ratio 1:1v/v, 1: 1:1v/v and 1:1:1:1v/v.

### Minimum inhibitory concentrations (MIC) by broth dilution method

MIC values were determined by a two-fold microdilution method to assess the antibacterial effects of herb-herb combinations according to Kudumela *et al*. (2018)**.** In brief, sterile, 96-well polystyrene microtiter plates was first preloaded with 50μL of Mueller Hinton broth in each well followed by the addition of 50μL of extract solutions into the first well of each row to make a total volume of 100μL. Each of the test sample materials was tested in duplicate. To the first well, the samples were mixed and 50μL was drawn from each well and transferred to the subsequent wells until the last wells. Then 50μL of the mixture from the last well was discarded. Thereafter, 50μL of the bacterial suspension equivalent to 0.5 MacFarland standard turbidity (1.5×106 CFU mL-1) was added to each well. An additional row containing 0.1mg/ml of gentamicin (50μL) was used as a positive control. Wells containing (50μL) solvent and bacteria only were used as negative controls. The plates were incubated at 37°C overnight. MIC was determined visually, whereby the lowest concentration without growth of bacteria was considered as the MIC.

### Fractional inhibitory concentration (FIC)

Checkerboard assay was employed to determine the Fraction Inhibitory Concentration (FIC) as described (Jain *et al.*, 2011)**.** FIC is determined by a methodology similar to that utilized for the determination of MIC, however modified so that it is useful to test the antibacterial activities of combinations of extracts (Meletiadis *et al.*, 2010)**.** The summation of fractional inhibitory concentration (ΣFIC) was calculated for each tested sample independently as specified in the following algebraic formula (Kudumela *et al.*, 2018).

FIC index = FIC *Cs* + FIC *Sg*

Where:


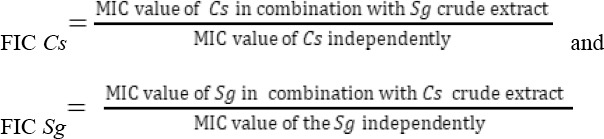


Where the combined effect, was interpreted as synergistic if the FIC index ≤0.5, additive if 0.5 > FIC Index < 4, or antagonistic if FIC Index ≥ 4. This interpretation follows the conventional model suggested by (Odds, 2003) and Kassim *et al.*, (2016).

888

## Results

### Antibacterial activity of individual extracts

The evaluations of antibacterial activities of individual extracts were conducted and the MIC of each extract was obtained as indicated in Table 1 and 2. The MIC values were interpreted based on classification criteria as follows; 0.05-0.5mg/mL strong activity, 0.6-1.5mg/mL moderate activity and above 1.5mg/mL weak activity (Sartoratto *et al.*, 2004). Among the crude extracts tested, methanol extracts of leaves, stem barks and root barks of *S. glaucescens* and *C. swynnertonii* inhibited the growth of gram-positive bacteria *S. aureus* and *E. faecalis* considerable with the lowest MIC values range 0.011 – 0.375mg/mL as shown in Table 1 and 2. Dichloromethane extracts of *S. glaucescens* and *C. swynnertonii* showed moderate antibacterial activity against Gram-positive bacteria tested with MIC values range 0.75mg/mL-1.5mg/mL. Furthermore, all extracts showed weak activity against Gram-negative bacteria (Tables1 and 2). However, gentamicin showed stronger antibacterial activity than the extracts tested (Tables1 and 2).

**Table 1:** Minimum inhibitory concentration (mg/mL) of individual crude extracts of *Commiphora swynnertonii* tested against selected bacteria

Extracts/Gentamicin	Minimum inhibitory concentration (mg/mL)				

	Gram-positive bacteria		Gram-negative bacteria		
	*S. aureus* ATCC 29213	*E. coli* ATCC 25922	*E. faecalis* ATCC 1559	*K. pneumoniae* ATCC 1145	*P. aeruginosa* ATCC 27853
*Cs*7D	0.37	3	0.75	3	3
*Cs*7M	0.09	3	0.37	3	3
*Cs*5D	0.75	3	1.5	3	3
Cs5M	0.18	1.5	0.75	3	3
*Cs*2D	1.5	3	0.37	3	3
*Cs*2M	0.04	3	0.37	3	3
Gentamicin	0.002	0.004	0.002	0.008	0.004

Key: D= Dichloromethane extract, M= Methanol extract, *Cs= Commiphora swynnertonii,* *Cs*7 *leaves* extracts, *Cs*5 stem bark extracts, *Cs*2 root bark extracts.

**Table 2:** Minimum inhibitory concentration (mg/mL) of individual crude extracts of *Synadenium glaucescens* tested against selected bacteria

Extract/Gentamicin	Minimum inhibitory concentration (mg/mL)				

	Gram-positive bacteria		Gram-negative bacteria		
	*S. aureus* ATCC 29213	*E. faecalis* ATCC 1559	*E. coli* ATCC 1559	*K. pneumoniae* ATCC 1145	*P. aeruginosa* ATCC 27853
*Sg*7D	0.75	0.75	3	3	1.5
*Sg*7M	0.37	0.37	3	3	0.75
*Sg*5D	0.37	0.75	3	3	3
*Sg*5M	0.02	1.5	3	3	3
*Sg*2D	0.02	0.37	3	3	1.5
*Sg*2M	0.01	0.02	1.5	3	1.5
Gentamicin	0.002	0.002	0.004	0.008	0.004

Key: D= Dichloromethane extract, M= Methanol extract, Sg=Synadenium glaucescens, Sg7=leaves extracts, Sg5=stem bark extracts, Sg2= root bark extracts

### Antibacterial activity of combined crude extracts and fractional inhibitory concentrations

The combination effects were evaluated with respect to MIC value of each crude extract against bacteria. In the combination of 1:1v/v, the extracts exhibited strong activity against Gram-positive bacteria *S. aureus* and *E. faecalis* with MIC values ≤0.5 (Table 3). These combinations include methanol extracts of *C. swynnertonii* leaves and stem barks of *S. glaucescens*, *C. swynnertonii* leaves and root barks of *S. glaucescens*, stem barks of *C. swynnertonii* and *S. glaucescens* leaves, stem barks of *C. swynnertonii* stem barks of *S. glaucescens*, stem barks of *C. swynnertonii* and root barks of *S. glaucescens*, root barks of *C. swynnertonii* and *S. glaucescens* leaves, root barks of *C. swynnertonii* and stem barks of *S. glaucescens*, and root barks of *C. swynnertonii* and root barks *S. glaucescens*.

**Table 3:** Minimum inhibitory concentration (mg/mL) of combined crude extracts from *Commiphora swynnertonii* and *Synadenium glaucescens* tested against selected bacteria

Combinations	Minimum inhibitory concentration (mg/mL)				

	Gram-positive bacteria		Gram-negative bacteria		
	*S. aureus* ATCC 29213	*E. faecalis* ATCC 1559	*E. coli* ATCC 25922	*K. pneumoniae* ATCC 1145	*P. aeruginosa* ATCC 27853
*Cs*7D+*Sg*7D	1.5	0.75	3	3	3
*Cs*7M+*Sg*7M	0.07	0.75	3	3	3
*Cs*7D+*Sg*5D	0.37	0.37	3	3	3
*Cs*7M+*Sg*5M	0.02	0.07	3	3	3
*Cs*7D+*Sg*2D	0.75	0.28	3	3	3
Cs7M+*Sg*2M	0.01	0.21	3	3	3
Cs7M+*Sg*7M+*Sg*5M	0.75	0.37	3	3	3
*Cs*7M*+Sg*7M*+Sg*5M*+Sg*2M	0.09	0.37	3	3	3
Cs5D+*Sg*7D	0.75	0.75	3	3	3
*Cs*5M+*Sg*7M	0.07	0.14	3	3	3
*Cs*5D+*Sg*5D	0.37	0.28	3	3	3
*Cs*5M+Sg5M	0.11	0.14	3	3	3
*Cs*5D+*Sg*2D	0.75	0.11	3	3	3
*Cs*5M+*Sg*2M	0.19	0.39	1.5	3	1.5
Cs5M+*Sg*7M+*Sg*5M	0.18	0.75	3	3	3
*Cs*5M*+Sg*7M*+Sg*5M*+Sg*2M	0.18	0.37	3	3	3
*Cs*2D+*Sg*7D	0.75	0.56	3	3	3
Cs2M+*Sg*7M	0.05	0.37	1.5	3	3
*Cs*2D+*Sg*5D	0.37	0.56	3	3	3
*Cs*2M+*Sg*5M	0.008	0.14	1.5	3	3
C*s2* D+*Sg*2D	0.75	0.18	3	3	3
*Cs*2M*+Sg*2M	0.01	0.05	0.75	3	3
Cs2M+*Sg*7M+*Sg*5M	0.09	0.18	3	3	3
*Cs*2M*+Sg*7M*+Sg*5M*+Sg*2M	0.09	0.04	3	3	3

Key: + = combination, D= Dichloromethane crude extracts, M= Methanol crude extracts, *Cs*7 *Commiphora swynnertonii* leaves extracts, *Cs*5 *Commiphora swynnertonii* stem bark extracts, *Cs*2 *Commiphora swynnertonii r*oot bark extracts, *Sg*7 *Synadenium glaucescens* leaves extracts, *Sg*5=*Synadenium glaucescens* stem bark extracts, *Sg*2 *Synadenium glaucescens* root bark extracts.

However, crude extracts combined in ratios 1:1:1 and 1:1:1:1v/v revealed moderate activity against *S. aureus* with MIC values range 0.6-1.5mg/mL (Table 3). Additionally, these combinations exhibited weak antimicrobial activity with MIC values above 1.5 mg/mL (Table 3) against the tested gram-negative bacteria *E. coli*, *K. pneumoniae* and *P. aeruginosa*. The FIC values were calculated and antibacterial effects were outlined in Table 4. In 1:1v/v combinations, One (1) synergistic effect observed in combination of methanol extracts of *C. swynnertonii* stem barks and *S. glaucescens* leaves against *S. aureus* (∑FIC 0.5) (Table 4). Other two synergistic effects were observed against *E. faecalis* in dichloromethane extracts of *C. swynnertonii* stem barks and *S. glaucescens* stem barks (∑FIC 0.5), and *C. Swynnertonii* root barks and *S. glaucescens* root barks with FIC index 0.3 (Table 4). Furthermore, three (3) antagonistic effects were observed in the combinations of dichloromethane leaves extract of *C. swynnertonii* and root barks of *S*. *glaucescens*, stem barks of *C. swynnertonii* and root barks of *S*. *glaucescens*, and root barks of *C. swynnertonii* and root barks of *S*. *glaucescens* against*. S. aureus* with FIC Index values 6, 19, and 38 *(*Table 4). In addition, other antagonistic effects were observed against *E. faecalis* in combinations of methanol leaves extract of *C. swynnertonii* and *S*. *glaucescens* leaves, stem barks of *C. swynnertonii*, and root barks of *S*. *glaucescens*, and leaves of *C. swynnertonii* and root barks of *S*. *glaucescens* with FIC Index values 10 and 19 (Table 4). The 1:1:1v/v and 1:1:1:1v/v combination ratios revealed antagonistic effects against *S. aureus* and additive effects against *E. faecalis* (Table 4). Moreover, the extracts in the combination ratio of 1:1v/v and 1:1:1v/v tested against Gram-negative bacteria revealed additive effects with FIC Index value 2 (Table 4), whereby the extracts in the combination ratio of 1:1:1:1:1v/v showed different antagonistic effects against Gram-negative bacteria with FIC Index values 4, 5, 6 and 8 (Table 4).

**Table 4:** Fractional inhibitory concentration Index (FIC Index) of combined crude extracts from *Commiphora swynnertonii* and *Synadenium glaucescens* tested against selected bacteria.

	Fractional inhibitory concentration (FIC) Index				
Combinations	Gram-positive bacteria		Gram-negative bacteria		
	*S. aureus* ATCC 29213	*E. faecalis* ATCC 1559	*E. coli* ATCC 25922	*K. pneumoniae* ATCC 1145	*P. aeruginosa* ATCC 27853
*Cs*7D+*Sg*7D	6.1	2.1	2	2	3
*Cs*7M+*Sg*7M	1.8	2.5	2	2	2
*Cs*7D+*Sg*5D	2	1.3	2	2	3
*Cs*7M+*Sg*5M	1.2	0.6	2	2	2
*Cs*7D+*Sg*2D	3	1.1	2	2	3
Cs7M+*Sg*2M	1.2	1.3	2	2	2
Cs7M+*Sg7* M+*Sg5* M	47.8	2.2	3	3	6
*Cs*7M+*Sg*7M+*Sg*5M+*Sg*2M	14.7	20.5	5	4	8
Cs5D+*Sg*7D	38.5	1.1	2	2	3
*Cs*5M+*Sg*7M	0.5	1.2	2.5	2	5
*Cs*5D+*Sg*5D	38	0.5	2	2	3
*Cs*5M+Sg5M	0.7	2.2	3	2	5
*Cs*5D+*Sg*2D	38.5	0.6	2	2	3
*Cs*5M+*Sg*2M	1.2	0.9	2	2	4.5
Cs5M+*Sg*7M+*Sg*5M	10.4	3.5	4	3	6
*Cs5* M+*Sg* 7M+*Sg* 5M+*Sg* 2M	28.4	20.4	5	4	8
*Cs* 2D+*Sg*7D	1.5	1.6	2	2	2
*Cs*2 M+*Sg*7M	2.2	3.4	1	2	3
*Cs* 2D+*Sg*5D	5.2	1.6	2	2	2
*Cs* 2M+*Sg*5M	19.2	19.8	1.5	2	2
Cs*2* D+*Sg*2D	1.4	0.3	1.5	2	3
*Cs2* M*+Sg*2M	2.2	10.6	2.2	2	3
*Cs*2M+*Sg*7M+*Sg*5M	69	1.1	3	3	6
*Cs*2M*+Sg*7M*+Sg*5M*+Sg*2M	15.9	2.2	5	4	8

Key: + = combination, D=Dichloromethane crude extracts, M=Methanol crude extracts, *Cs*7 *Commiphora swynnertonii* leaves extract, *Cs*5 *Commiphora swynnertonii* stem bark extracts, *Cs*2 *Commiphora swynnertonii* root bark extracts, *Sg*7= *Synadenium glaucescens* leaves extracts, *Sg*5=*Synadenium glaucescens* stem bark extracts, *Sg*2 *Synadenium glaucescens* root bark extracts

## Discussion

### Antibacterial activity of individual and combined crude extracts

Herbal medicines are normally prepared either singly or in combination with several plant species (Vuuren and Viljoen, 2011). In this study, crude extracts from leaves, stem barks, and root barks of *C. swynnertonii* and *S. glaucescens* were screened for antibacterial properties both individually and in combinations against selected bacteria. The findings of this study for the individual plant parts of *C. swynnertonii* are in agreement with previous studies reported by Bakari *et al*. (2011) and Mkangara *et al*. (2014).

Bakari *et al*. (2011) confirmed antibacterial and anti-Candida activities of the methanol extracts of the leaves from stem and root barks of *C. swynnertonii, and Makangara et al. (2014)* reported the activity of the same parts of the plant against pathogenic bacterial and fungal species. Hence, the results of this study together with those previously reported supporting the traditional uses of these plant parts for the management of bacterial and fungal infections.

Furthermore, a previous study conducted by Max *et al*. (2014) for the crude root extract of *S. glaucescens* reported antibacterial activity against *S. aureus* and moderate activity against *P. aeruginosa*. Similarly, in the current study individual methanol extracts of the parts of *S. glaucescens* showed strong activity against *S. aureus* and *E. faecalis*.

In this study, however, the individual extracts of these plant parts displayed weak activity against Gram-negative bacteria tested. The difference in susceptibility for Gram-positive bacteria and Gram-negative-bacteria may be associated with differences in their cell wall structure. Gram-negative bacteria are reported to be more resistant due to impermeability/efflux of their outer membrane/cell wall which acts as a barrier to many environmental substances including herbal drugs or antibiotics (Rawat and Nair, 2010).

Moreover, this study reports the antibacterial effects of combined crude extracts of *S. glaucescens* and *C. swynnertonii*. It is clear from Table 4 that there is a greater antibacterial activity in some combined extracts than individual extracts. The combined extracts which showed synergistic effects may be promising alternatives for antibacterial therapy in the future, and their effects should be investigated further. Several synergistic effects of herb-herb combinations done in different plants have been reported in previous studies. Rapper *et al*. (2016) substantiated this point of synergy in the combinations of *Schkuhria pinnat*a and *Commelina africana, Dombeya rotundifolia*, and *Schkuhria pinnata* against *P. aeruginosa* with ∑FIC values ≤ 0.5. Another synergic effects were demonstrated in the combinations of *Bidens pilosa* and *Leonotis nepetifolia* extracts against *Candida albicans (Mbunde et al., 2019)*. The synergistic effects observed in some combinations (Table 4) imply that there is an increase in antibacterial activity of the combined crude extracts against Gram-positive bacteria as a result of the summation of their individual effects.

However, in this study additive effects were also demonstrated in several combinations (Table 4). This effect occurs when the activity of the combined extracts is equivalent to the sum of the activity of each extract when used individually (Adams *et al.*, 2006). This effect signifies that the biological actions of the combined extracts interact with similar molecular targets or metabolic pathways (Vuuren and Viljoen, 2011). Antagonistic effects were also observed in some combinations against the tested bacteria (Table 4). This indicates that, the extracts have conflicting effect that may block or reduce the effectiveness of one or both extracts. Usually, this type of effect is discouraged for therapeutic application (Bassolé and Juliani, 2012).

## Conclusion

Combined extracts of *S. glaucescens* and *C. swynnertonii* have additive effects against gram-positive bacteria tested. Further, combined extracts of root barks of *C. swynnertonii* and stem barks of *S. glaucescens* have synergistic effect against gram-positive bacteria tested, suggesting that it can be advantageous to combine such extracts to form their products.

Therefore, based on the combinations which showed synergistic effects against some of the tested bacteria, this study provides promising alternative herbal antimicrobials from plants. However, it is recommended that further studies on the combinations that showed synergistic effects should be carried out on their toxicity and mode of action to optimize their use.

### Conflict of interest statement

The authors declare that they have no conflict of interest associated with this study.

**Abbreviations:**
MIC=: Minimum Inhibitory Concentration,
FIC=: Fractional Inhibitory Concentration,
ATCC=: AmericanType Culture Collection,
DMSO=: Dimethyl sulfoxide,
CFU mL^-1^ =: Colon Forming Unit per milliliter,
mg/mL=: Milligram per milliliter,
S. aureus=: Staphylococcus aureus,
E. faecalis=: Enterococcus faecalis,
E. coli=: Escherichia coli,
K *pneumonia*=: *Klebsiella pneumonia,*
P. aeruginosa =: *Pseudomonas aeruginosa*
+ =: combination,
DCM/D=: Dichloromethane crude extracts,
MeOH/M=: Methanol crude extracts,
*Cs*7=: *Commiphora swynnertonii* leaves extracts,
*Cs*5=: *Commiphora swynnertonii* stem bark extracts,
*Cs*2=: *Commiphora swynnertonii r*oot bark extracts,
*Sg*7=: *Synadenium glaucescens* leaves extracts,
*Sg*5=: *Synadenium glaucescens* stem bark extracts,
*Sg*2=: *Synadenium glaucescens* root bark extracts.
